# On the Use of Aqua Frigida in Scalds, in Epidemic Ophthalmia, and the Effects of Cold on the Human Body

**Published:** 1805-09-01

**Authors:** Ralph Cuming

**Affiliations:** His Majesty's Ship Pegasus


### On the Use of Aqua Frigida in Scalds, in Epidemic Ophthalmia, and the Effects of Cold on the Human Body. By Ralph Cuming, M. D. *of His Majesty's Ship Pegasus.*

*To the Editors of the Medical and Physical Journal.*

Gentlemen,

IMPRESSED with an idea that time can never be better employed, than when devoted to alleviate the sufferings of our fellow creatures; I have to request, that you will be pleased to insert the following paper in your very useful Miscellany, which I hope may have some share in being conducive towards the attainment of so desirable an end.

On the treatment of scalds and burns, there seems to have been great diversity of opinion, if one may be allowed to judge, from the different modes of cure which have been adopted by medical practitioners. The oleum lini sine igne, was formerly in high repute, and generally speaking, had, and still is allowed to have, by many, the precedence as a remedy; in recent cases, some mix with it aq. litharg. acetata, aq. calcis, &c. Vinegar used to be in high estimation at Edinburgh. All of these, and many other applications, I have seen tried; but as far as my observation leads me, they are not nearly so beneficial as the plan which I have for some time accustomed myself to; and I may say, most successfully; if speedy relief from pain, and a quick and easy cure, are desiderata claiming the attention either of the patient or medical attendant. I have read and heard much about the efficacy and superior power of ol. terebinthinæ in those cases, but all that I know of it is from report, for I never could make up my mind to use it, conceiving that it was a harsh remedy, and such an one as was calculated to produce exquisite *torture;* on this ground alone have I rejected it, as it has the appearance of applying one species of fire to extract another.

Aqua

Aqua frigida is my favourite remedy; in the last ship I served in, the Malabar, one of the Serjeants of Marines, as well as many others, were scalded; but as this was the worst case, I mention it in preference; part of the leg, the whole of the ankle, and upper as well as lower part of the foot, were vesicated. He was ordered to place his foot and ankle in a bucket full of cold water, which immediately relieved him from the excruciating pain he complained of; and when it became necessary to renew the water, which was frequently done when it acquired a certain degree of heat, imparted to it from the inflamed limb, he was extremely anxious for a fresh supply, for the colder the water was, the more comfortable he said he felt the part affected.

At night, nurses were appointed to attend him, with directions to apply linen rags dipped in water, and to renew them whenever they became warm; and this mode of treatment was persevered in until the inflammation subsided. Some of the vesications were very large, and by way of experiment I cut one of them through its whole extent, but found this part more difficult to heal than the others, from which the serum was evacuated by small punctures.

But after the inflammation is repelled, or I would rather say, superabundant heat is extracted, it is evident that something still remains to be done, as loss of continuity, or destruction of organization, must always take place, in a ratio proportionate to the extent of the injury sustained; which, sometimes, is so slight, as only to affect the epidermis and rete mucosum; but when the cutis vera is affected, a suppurative process must take place before the cure can be completed, and I found it necessary in this instance to apply emollient and warm cataplasms for that purpose; after which the cerat. lapidis calaminaris completed the cure in a few days, which would in all probability have been protracted for several weeks, had not the inflammation been speedily subdued by the refrigerating method.

It will appear from analogy and observation derived from our knowledge of the cure of all inflammatory complaints, that the above modus curandi is not the chimerical product of theoretical disquisition, or fanciful hypothesis; no, quite the reverse, for it has the support of successful experience, deduced from the principles of natural philosophy. And by way of illustration I shall beg leave to mention, that, lately, whilst cruizing off the Dutch Coast,

 several several of our men were affected with ophthalmia epidemica, occasioned, as I suppose, from the cutting easterly winds, which at that time prevailed; their eyes were much blood-shot, and the tunica adnata so much distended in two or three instances, as to protrude beyond the edges of the tarsi, yet almost all of them recovered in the course of three or four days, without having recourse to any *general* treatment; aq. frigida incessantly applied, effected a cure tute, celeriter et jucunde.

The effect of cold water on the human body is a subject which, if rightly understood, would throw great light on therapeutics, and so illuminate this path of medical science, as to enable the physician to divest his mind of much anxiety and diffidence; but when things of importance, from a want of knowledge respecting their action, are not properly understood, every one is left to form his own opinion, and wander in the mazes of doubt and uncertainty; and that theory which is most plausible, supported by reasoning, deduced from observation and well attested facts, will be generally received. Hence arises that difference of opinion, which prevails among medical authors. Cullen says, "Cold is *always* more or less *directly* sedative; but it is equally manifest, that in *certain* circumstances cold proves a stimulant to the living body, and particularly to the sanguiferous system." This account of it is both inconsistent and unsatisfactory.

Brown's opinion is, that it always acts as a debilitating power, and observes, "Because cold is so efficacious a remedy in the small-pox, it clearly follows that the use of cold should be extended to the whole range of predisposition, the whole circle of diseases depending upon sthenic diathesis."

Cold, according to my opinion, may be said to have a twofold effect, for its action on the human body must depend upon the mode of its application, and will be modified agreeably to the temperature of the body. For instance, cold is always grateful to a person when oppressed with heat, giving vigour to the relaxed frame; it acts in a manner diametrically opposite, when the body is cold, and circulation languid; and is the source of a variety of diseases. So that to say cold is either sedative or stimulant, or immediately debilitating, does not appear to me to convey a correct notion of its real properties and effects. Let us suppose that the heat of the body in a state of good health is at 90°; if cold should be applied sufficient to reduce it to 60°, the constitution will suffer in proportion of to the time of its application, and susceptibility of the body. On the other hand, we shall imagine that the heat rises to 110°, and the application of cold reduces it to the standard above at 90°, it is evident that in the one instance it will produce debilitating, in the other tonic effects.

What I have said on this subject may be considered as being too concise to render it worthy of much attention, but facts will ever have a proper influence on the mind of the man, who, from the purest motives, is in the habit of contributing his mite of knowledge to the general stock; who, fearless of reproach, and buoyed up by conscious integrity,

> Can laugh at envy, nor foul censure fears,
> Nor dreads the fabric that vile Mendax rears.

*Harwich, August,* 4, 1805.

